# Prediction of post-Schroth Cobb angle changes in adolescent idiopathic scoliosis patients based on neural networks and surface electromyography

**DOI:** 10.3389/fbioe.2025.1570022

**Published:** 2025-05-14

**Authors:** Shuguang Yin, Jiangang Chen, Peng Yan

**Affiliations:** ^1^ Department of Clinical Medicine, Suzhou Vocational Health College, Suzhou, China; ^2^ College of Physical Education and Sport, Beijing Normal University, Beijing, China; ^3^ Department of Rehabilitation Medicine, Suzhou Municipal Hospital, Suzhou, China

**Keywords:** adolescent idiopathic scoliosis (AIS), Cobb angle, Schroth exercises, neural networks, TCN-LSTM hybrid model, surface electromyography (SEMG)

## Abstract

**Introduction:**

To develop a temporal-convolutional-LSTM (TCN-LSTM) hybrid model integrating surface electromyography (sEMG) signals for forecasting post-Schroth Cobb angle progression in adolescent idiopathic scoliosis (AIS) patients, thereby offering accurate feedback for personalized treatment.

**Methodology:**

A total of 143 AIS patients were included. A systematic Schroth exercise training program was designed. sEMG data from specific muscles and Cobb angle measurements were collected. A neural network model integrating Temporal Convolutional Network (TCN), Long Short-Term Memory (LSTM) layers, and feature vectors was constructed. Four prediction models were compared: TCN-LSTM hybrid model, TCN, LSTM, and Support Vector Regression (SVR).

**Results:**

The TCN-LSTM hybrid model demonstrated superior performance, with Cobb angle-Thoracic (Cobb Angle-T) prediction accuracy reaching R^2^ = 0.63 (baseline) and 0.69 (Week 24), achieving overall R^2^ = 0.74. For Cobb angle-Lumbar (Cobb Angle-L), accuracy was R^2^ = 0.61 (baseline) and 0.65 (Week 24), with overall R^2^ = 0.73. The SVR model showed lowest performance (R^2^ < 0.12).

**Conclusion:**

The TCN-LSTM hybrid model can precisely predict Cobb angle changes in AIS patients during Schroth exercises, especially in long-term predictions. It provides real-time feedback for clinical treatment and contributes to optimizing treatment plans, presenting a novel prediction approach and reference basis for evaluating the effectiveness of Schroth correction exercises in AIS patients.

## 1 Introduction

Adolescent Idiopathic Scoliosis (AIS) is a common spinal deformity during puberty, characterized by spinal curvature and rotation (Peng et al., 2020). It affects 2%–3% of adolescents, with most cases being idiopathic (Kuznia et al., 2020). While genetic or congenital factors contribute to some cases, the majority lack clear physiological or environmental triggers. Acquired factors like poor posture, inactivity, and obesity may worsen spinal curvature (Weinstein et al., 2008; Altaf et al., 2013). Without timely intervention, AIS can progress, impairing physiological function and daily activities (Cheng et al., 2015). AIS impacts adolescents’ health in multiple ways: (1) Altered physical appearance may lead to low self-esteem (Horne et al., 2014); (2) Severe curvature compromises respiratory function and organ health (Reamy and Slakey, 2001; Lonstein, 1994); (3) Progressive deformity limits mobility and causes chronic pain (Schreiber et al., 2014; Bleck, 1991); (4) Psychological stress, including anxiety and depression, is prevalent due to physical and social challenges (Ma et al., 2023). Early diagnosis and intervention are thus critical. Currently, AIS severity is assessed via Cobb angle measurements from spinal radiographs (Mohamed and Yousef, 2021). The Cobb angle, calculated from vertebral inclination lines, classifies curvature as mild (<25°), moderate (25°–45°), or severe (>45°) (Jin et al., 2022). However, this method has limitations: (1) Operator-dependent errors and radiation exposure risks (Pasha et al., 2021); (2) Inability to capture spinal rotation (Greiner, 2002); (3) Time-consuming processes hinder real-time monitoring. A rapid, accurate alternative for tracking AIS progression is urgently needed.

Electromyography (EMG) detects muscle activity through bioelectrical signals, providing insights into muscle function and contraction timing (Farina, 2006). In scoliosis patients, EMG reveals asymmetric muscle activation patterns, offering valuable information about disease mechanisms and progression (Wang et al., 2022). Surface EMG (sEMG) enables non-invasive monitoring of muscle activity, showing promise for assessing motor function in AIS (Zhang et al., 2024). However, EMG applications in AIS remain limited due to challenges in data analysis sensitivity and equipment requirements. Deep learning approaches, especially Temporal Convolutional Networks (TCN) and Long Short-Term Memory (LSTM) networks, excel at processing sequential biomedical data (Dolan et al., 2024). TCN effectively models long-term dependencies through convolutional layers, while LSTM addresses the vanishing gradient problem in recurrent networks (Ruiz-Garcia et al., 2021). These methods have proven successful in ECG and EEG analysis (Zhang et al., 2019; Currie et al., 2019), yet their application to scoliosis research, particularly in combination with EMG data, remains underdeveloped. Integrating neural networks with EMG could enable more accurate tracking of disease progression and personalized treatment strategies.

Current treatments for adolescent idiopathic scoliosis (AIS), including physiotherapy, bracing, and surgery, demonstrate effectiveness but have limitations (Kocaman et al., 2021; Yagci and Yakut, 2019). For mild to moderate cases, conventional bracing and surgery may negatively impact long-term quality of life (Weinstein et al., 2013), while physiotherapy often requires extended treatment periods with variable outcomes (da Silveira et al., 2022). Schroth exercises offer a non-invasive alternative, focusing on spinal symmetry through targeted positioning, breathing techniques, and postural correction (Ceballos-Laita et al., 2023). This approach not only improves Cobb angles and spinal rotation but also enhances muscle endurance and maintains spinal structure (Mohamed and Yousef, 2021). Importantly, Schroth exercises also provide psychological benefits by boosting self-confidence and reducing anxiety (Kuru et al., 2016), making them a valuable treatment option for AIS.

This study introduces an innovative approach combining neural networks (TCN-LSTM) with EMG data to improve AIS management. Our primary goal is to develop an accurate prediction model for scoliosis progression during Schroth therapy by analyzing sEMG signals and multi-dimensional clinical indicators (spinal rotation, Cobb angle, muscle endurance). This integration provides: (1) a novel diagnostic perspective through EMG pattern analysis; (2) an objective assessment tool for Schroth exercise efficacy; and (3) data-driven support for personalized treatment plannin. The proposed method addresses current research gaps and offers clinically significant technical advancements for early AIS intervention.

## 2 Methods

### 2.1 Research subjects

A total of 143 adolescent idiopathic scoliosis (AIS) patients were included in this study, all recruited from the outpatient department of Hospital. The age range of the patients was from 10 to 18 years old, with an average age of (14.6 ± 2.7) years. This age group is the high-incidence stage of AIS, during which the spine is still in the process of growth and development and has relatively high plasticity. The initial Cobb angle of the included patients ranged from 10° to 40°, with an average Cobb angle of (17.9 ± 5.9)°. This range covered mild to moderate scoliosis, and for such patients, conservative treatment is usually the main approach, and the Schroth exercise therapy is a commonly used conservative treatment method. All patients were diagnosed through detailed physical examinations, full-length spinal X-ray films, CT scans, and other examinations. Patients with scoliosis caused by other reasons such as congenital spinal deformities, neuromuscular diseases, and trauma were excluded to ensure that the research subjects were patients with simple AIS. In addition, none of the patients had underlying diseases such as severe back pain, severe cardiopulmonary dysfunction, or cognitive dysfunction that could affect exercise training and data collection. They were in generally good physical condition and were able to cooperate to complete the Schroth exercises and related examination tests. Before the study was carried out, all patients and their families fully understood the purpose, process, and potential risks of the study, and voluntarily signed the informed consent form. The research protocol was reviewed and approved by the Ethics Committee and strictly adhered to the Declaration of Helsinki.

The sample size of this study was 143 cases, which was determined by referring to the sample sizes of previous similar studies and based on the statistical power analysis method. During the preliminary literature review, it was found that the sample sizes of most electromyographic and biomechanical studies related to the rehabilitation treatment of AIS patients were mostly around 20–50 cases. AIS patients are a relatively scarce group, so the sample collection was limited. To address the issue of small samples, it was ensured that each patient completed multiple exercises to construct a larger data set.

### 2.2 Schroth exercise intervention program

**FIGURE 1:**
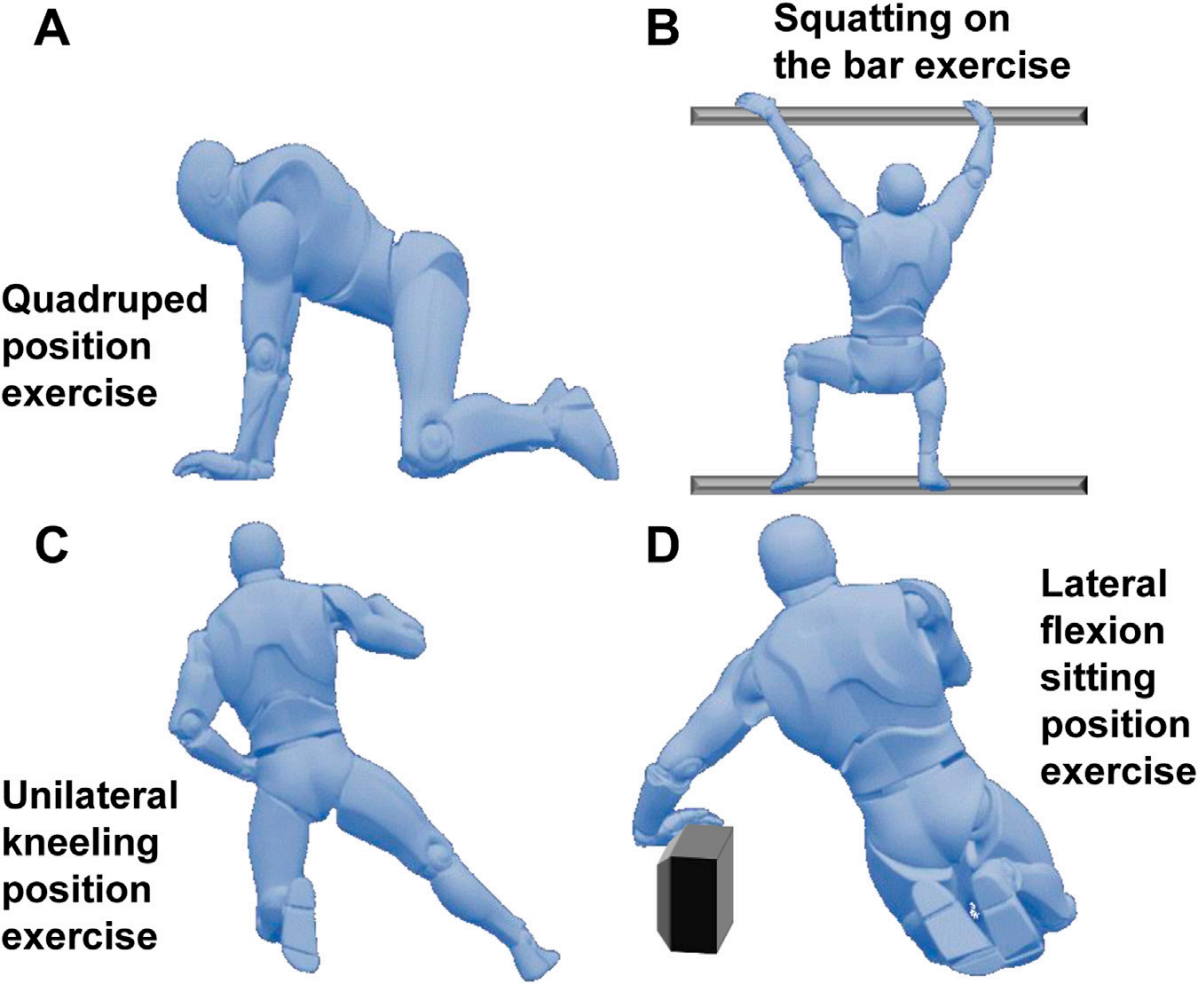
Schroth exercise intervention program. **(A)**: Quadruped position exercise; **(B)**: Squatting on the bar exercise; **(C)**: Unilateral kneeling position exercise; **(D)**: Lateral flexion sitting position exercise.

Based on the Schroth therapy system and combined with the individual spinal scoliosis characteristics of the patients, a systematic exercise training program was formulated for the patients, which included four typical Schroth exercises (See Figure 1):

• Quadruped position exercise: The patient placed their hands and knees on the ground, keeping the spine naturally extended, avoiding excessive collapse or arching. The head hung down naturally, and the eyes looked at the ground. On the basis of this position, the patient was guided to perform stable breathing, with the focus on activating core stabilizing muscle groups such as the erector spinae and multifidus muscles to enhance the spine’s control ability in the sagittal, coronal, and horizontal planes. This position was maintained for 20 s.
• Squatting on the bar exercise: The patient stood on a specially made bar close to the ground with feet shoulder-width apart, grasped the upper bar with both hands, and slowly squatted until the thighs were parallel to the ground. During the process, the spine was kept straight, avoiding leaning forward or backward, and the knees did not exceed the toes. The bar was used to assist in maintaining body balance, reducing the pressure on the waist, and maintaining spinal stability through the tightening of the core muscle groups to enhance the spine’s tolerance to vertical pressure. The downward position was maintained for 20 s.
• Unilateral kneeling position exercise: The patient knelt on one knee, extended the other leg to the side, placed both hands on the sides of the body, adjusted the pelvis to a horizontal position with the thigh on the kneeling side as support, and kept the spine extended to avoid exacerbating the scoliosis and convexity on the scoliosis side. On this stable basis, breathing was adjusted, and the imbalance of muscle strength on both sides of the spine was corrected to promote the spine’s return to the midline. This position was maintained for 20 s.
• Lateral flexion sitting position exercise: The patient sat with the buttocks placed on the heels of the feet and slowly tilted the body to one side. During the process, the pelvis was kept from twisting, and the hand on the tilted side could use a support block to assist in maintaining balance. This position was maintained for 20 s.

Before the formal collection of exercise data from the patients, professional rehabilitation therapists provided each patient with 30 min of action standard training every day for 3 days to ensure that the patients mastered the exercise skills proficiently. Each movement was performed in six sets, with 10 repetitions in each set, and a 1-min rest between each repetition. The training plan was carried out 3 times a week, with each session lasting 30 min and lasting for 24 weeks. During the exercise process, the therapists supervised the whole process, corrected the patients’ incorrect movements in a timely manner, and ensured the quality of the exercise and the validity of the data.

### 2.3 Surface electromyography (sEMG) signal acquisition and processing

**FIGURE 2:**
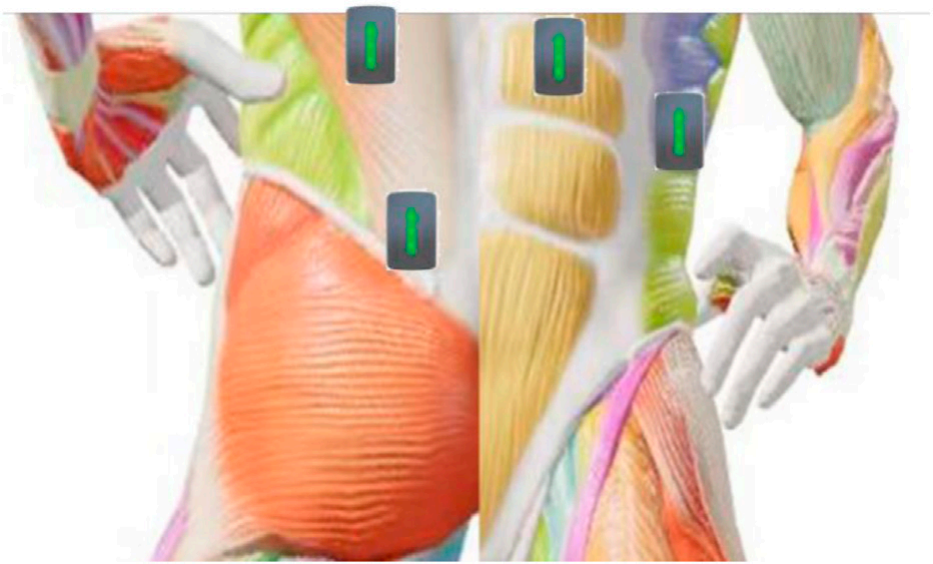
Placement of the EMG.

The sEMG sensors produced by the American DELSYS Company were used for surface electromyography signal acquisition. Based on the anatomical positions of the paraspinal muscles and their association with spinal scoliosis, the bilateral erector spinae (L4 - L5 segments), multifidus muscles, rectus abdominis, and external oblique muscles were selected as the sites for electromyography signal acquisition, with a total of eight muscles (See Figure 2). The positions for electromyography acquisition were arranged according to the SENIAM manual. Before attaching the electrode patches, the skin was wiped with alcohol cotton balls to reduce skin resistance and ensure good contact between the electrodes and the skin. The patches were attached parallel to the direction of the muscle fibers at the belly of the selected muscles, and the sampling frequency of the sensors was set to 1,000 Hz.

Before performing each Schroth exercise, the participants relaxed in a resting state for 3 min. Subsequently, during the exercise process, continuous electromyography signals were continuously collected during each movement. Only the electromyography signals during one training session were collected for each movement, and generally, a random signal among the 3rd to 8th repetitions of the second set of each movement was selected to ensure that muscle fatigue did not affect the signal quality. Each participant performed each movement once. Since there were four movements in total, 572 sample data were generated for each movement from 143 participants each time. Electromyography was measured at the 0th week, 8th week, 16th week, and 24th week. A total of 2,288 electromyography data were collected.

In this study, a fourth-order Butterworth band-pass filter was adopted. The lower cutoff frequency was set to 20 Hz, which could filter out low-frequency motion artifacts, such as slow potential changes caused by overall body shaking and respiratory movements. The upper cutoff frequency was set to 400 Hz, which could effectively remove high-frequency noise.

The root mean square value (RMS) was extracted from the filtered sEMG signals. The calculation formula is:

$$RMS=\sqrt{\frac{1}{N}\sum_{i=1}^{N}x_{i}^{2}}$$

where N is the number of signal sampling points, and $x_{i}$ is the electromyography signal value at the i-th sampling point.

And the paraspinal muscle symmetry index (PMSI) was calculated as $PMSI=\frac{RMS_{\text{convex}\;}}{RMS_{\text{concave}\;}}$ . A PMSI close to 1 indicated high symmetry of the paraspinal muscle. A PMSI<1 indicated that $RMS_{\text{concave}\;}$ was greater than $RMS_{\text{convex}\;}$ , and a PMSI>1 indicated that $RMS_{\text{convex}\;}$ was greater than $RMS_{\text{concave}\;}$ of the scoliotic curve.

These two features were calculated as the basic parameters for descriptive statistics and the basic information input into the neural network. All the remaining filtered electromyography signals were input into the neural network as time series data as a whole. The neural network adopted in this study could independently extract the time-domain and frequency-domain information from the electromyography signals.

### 2.4 Dependent variable measurements

#### 2.4.1 Spinal scoliosis angle (Cobb method)

The Cobb method was utilized to assess the degree of spinal curvature in both thoracic and lumbar regions. It is widely acknowledged as the gold standard for monitoring the progression of spinal scoliosis. The Cobb angles (in degrees) were obtained from standard anteroposterior standing full-spine X-ray films, with separate measurements performed for the thoracic (Cobb Angle-T) and lumbar (Cobb Angle-L) regions. X-ray examinations were performed in the posterior-anterior and lateral positions, with the patient in the anatomical standing position. The spinal scoliosis angles at both regions were measured independently at baseline and at the end of the 24-week period.

#### 2.4.2 Rotation angle (scoliometer)

The Bunnell scoliometer and Adam’s forward - bending test were employed to evaluate the trunk rotation angle (ART). The patient was required to bend forward, and the trunk rotation angle (the angle between the horizontal plane and the plane across the posterior part of the trunk) was measured using the apical vertebra of the curve. In cases of double scoliosis, ART was measured in the two most prominent regions, namely, the thoracic and lumbar regions. The rotation angle was measured at the 0th, 8th, 16th, and 24th weeks.

#### 2.4.3 Back muscle endurance

The Biering - Sorensen test (BST) is an effective and reliable method for measuring extensor muscle endurance. This test is used to evaluate the isometric endurance of the back muscles. To conduct this test, the patient lies face - down on an examination table and maintains the trunk in an extended position for as long as possible. The test concludes when the patient can no longer maintain the correct posture or when 240 s have elapsed. Back muscle endurance was measured at the 0th, 8th, 16th, and 24th weeks.

#### 2.4.4 Quality of life

The SRS - 22r questionnaire was applied to assess the quality of life (QoL). The SRS - 22r questionnaire is a scoliosis - specific quality - of - life questionnaire that evaluates five domains: function, pain, self - image, mental health, and satisfaction (with 5 questions in each domain except for treatment satisfaction, which contains 2 questions). Quality of life was measured at the 0th, 8th, 16th, and 24th weeks.

### 2.5 Neural network model construction

This study utilized Python’s TensorFlow framework to develop a hybrid deep learning model that combines a Temporal Convolutional Network (TCN), Long Short-Term Memory (LSTM) layers, and feature vectors to predict Cobb angle and other dependent variables. The TCN module was designed to extract local patterns and short-term features from time-series data, while the LSTM module captured global patterns and long-term dependencies. The feature vector component was responsible for extracting basic patient characteristics (e.g., BMI, gender). The model architecture consisted of multiple 1D convolutional layers, LSTM layers, and fully connected layers.

#### 2.5.1 1D convolutional layer

The formula for the 1D convolutional layer is as follows:

$$y=\sigma \left(W*x+b\right)$$

where:

• y : Output of the convolutional operation,
• σ : Activation function,
• W : Convolutional kernel weights,
• * : Convolution operation,
• x : Input,
• b : Bias term.

The 1D convolutional layers serve as the foundational blocks of the TCN, allowing the model to identify local temporal dependencies and extract short-term patterns in the time-series data.

#### 2.5.2 LSTM layer

The LSTM layer equations are defined as follows:

$$\begin{array}{c} f_{t}=\sigma \left(W_{f}\cdot \left[h_{t-1},x_{t}\right]+b_{f}\right)\\ i_{t}=\sigma \left(W_{i}\cdot \left[h_{t-1},x_{t}\right]+b_{i}\right)\\ {\tilde{C}}_{t}=\tanh\left(W_{C}\cdot \left[h_{t-1},x_{t}\right]+b_{C}\right)\\ C_{t}=f_{t}\cdot C_{t-1}+i_{t}\cdot {\tilde{C}}_{t}\\ o_{t}=\sigma \left(W_{o}\cdot \left[h_{t-1},x_{t}\right]+b_{o}\right)\\ h_{t}=o_{t}\cdot \tanh\left(C_{t}\right) \end{array}$$

where:

• $f_{t}$ : Forget gate,
• $i_{t}$ : Input gate,
• ${\tilde{C}}_{t}$ : Candidate cell state,
• $C_{t}$ : Current cell state,
• $o_{t}$ : Output gate,
• $h_{t}$ : Current hidden state,
• $W_{f},W_{i},W_{C},W_{o}$ : Weight matrices for the forget gate, input gate, candidate cell state, and output gate, respectively,
• $b_{f},b_{i},b_{C},b_{o}$ : Bias vectors for each respective gate,
• σ : Sigmoid activation function,
• tanh⁡ : Hyperbolic tangent activation function,
• $x_{t}$ : Input at time step ttt,
• $h_{t-1}$ : Hidden state at the previous time step,
• $C_{t-1}$ : Cell state at the previous time step.

The LSTM layer allows the model to maintain long-term dependencies, making it particularly effective for sequential data like sEMG signals.

#### 2.5.3 Fully connected layer

The formula for the fully connected layer is:

$$y=\sigma \left(Wx+b\right)$$

where:

• y : Output,
• σ : Activation function,
• W : Weight matrix,
• x : Input,
• b : Bias vector.

The fully connected layer combines features extracted by earlier layers to produce the final prediction output.

#### 2.5.4 Model architecture

**FIGURE 3:**
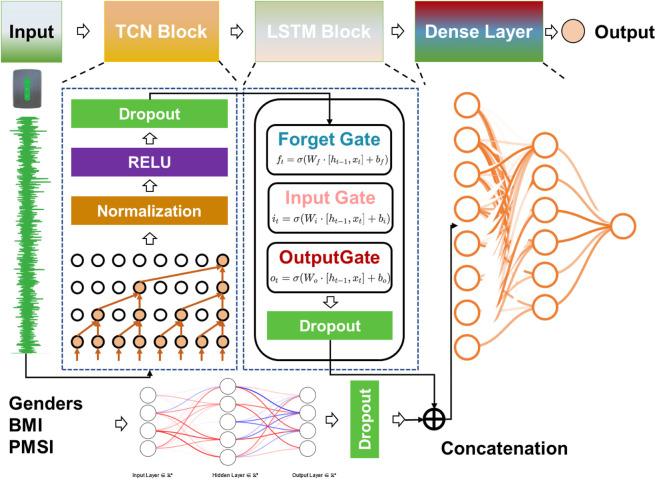
Architecture of neural networks.

The model architecture was composed of four modules in sequence (See Figure 3):

1. TCN Module: This module consisted of multiple 1D convolutional layers, batch normalization layers, and dropout layers, with the ReLU activation function used for non-linearity.
2. LSTM Module: This module included multiple LSTM layers to capture temporal dependencies and long-range relationships.
3. Fully Connected Layer: This layer processed non-time-series features such as gender, BMI, and multi-measurement PMSI data to generate a fixed-size feature vector.
4. Output Layer: The time-series features and non-time-series feature vectors were concatenated, and higher-order features were extracted through additional fully connected layers to produce the final predictions.

**FIGURE 4:**
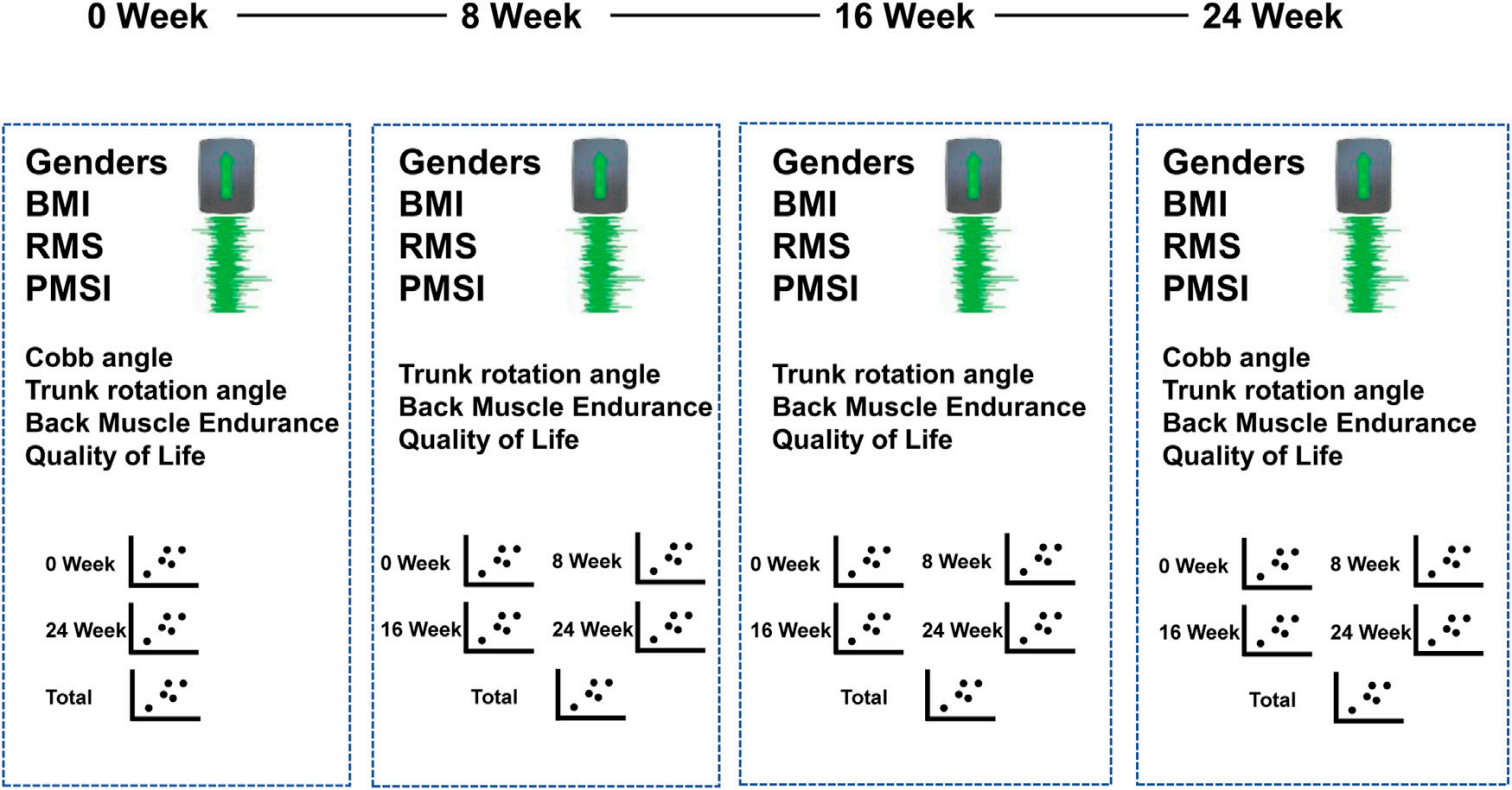
Selection of predictive variables.

The model predicted the following (See Figure 4):

• For Cobb angle: Input included sEMG signals and feature vectors from week 0 and week 24. The output was the Cobb angle at week 0 and week 12, providing a total of 186 samples.
• For rotation angle, back muscle endurance, and quality of life: Input included sEMG signals and feature vectors from weeks 0, 8, 16, and 24. The output was the dependent variables at these time points, providing a total of 572 samples.

#### 2.5.5 Optimization and training

The Adam optimizer was employed to dynamically adjust the learning rate and accelerate model convergence. To prevent overfitting, an early stopping strategy was implemented, halting training if the validation loss did not improve for 10 consecutive epochs. The model was trained for a maximum of 100 epochs.

#### 2.5.6 Performance evaluation

The trained model was evaluated on the test set using the following metrics:

1. Root Mean Square Error (RMSE): This metric measured the average deviation between predicted and true values. The formula is:
$$\text{RMSE}=\sqrt{\frac{1}{n}\sum_{i=1}^{n}{\left(y_{i}-{\hat{y}}_{i}\right)}^{2}}$$
  where:
• n : Number of samples,
• $y_{i}$ : True value,
• ${\hat{y}}_{i}$ : Predicted value.

2. Coefficient of Determination (R^2^): This metric quantified the goodness of fit of the model. The formula is:
$$R^{2}=1-\frac{\sum_{i=1}^{n}{\left(y_{i}-{\hat{y}}_{i}\right)}^{2}}{\sum_{i=1}^{n}{\left(y_{i}-\bar{y}\right)}^{2}}$$
  where:
• $\bar{y}$ : Mean of the true values.

Both RMSE and R^2^ were used to assess model accuracy and reliability in predicting Cobb angles and other dependent variables.

#### 2.5.7 Model training strategy

The dataset was randomly split into a 7:3 ratio for training and testing. To identify the optimal hyperparameter combination, a combination of grid search and random search was utilized:

• TCN Module: Hyperparameters included dilation rates (2, 4, 8, 16, 32), filter size (3), and dropout rates (0.1–0.5).
• LSTM Module: Hyperparameters included the number of neurons per layer (128, 256, 512) and dropout rates (0.1–0.5).
• Learning Rate: Initial range of 0.0001–0.01 with a step size of 0.0005.
• Batch Size: Fixed at 16.
• Fully Connected Layer Nodes: Explored configurations of 128, 64, and 32 nodes.

Exhaustive trials were conducted with all possible hyperparameter combinations. The optimal configuration was determined by comparing validation accuracy and loss across different combinations, ensuring faster convergence and higher prediction accuracy.

#### 2.5.8 Model validation

To ensure robust and reliable generalization, three-fold cross-validation was implemented:

1. The preprocessed dataset was randomly split into three mutually exclusive subsets: A, B, and C.
2. In the first iteration, subset A was used as the test set, while subsets B and C were combined as the training set.
3. In the second and third iterations, subsets B and C were sequentially used as the test set, with the remaining subsets forming the training set.

Performance metrics such as accuracy and RMSE were averaged across the three folds to obtain a comprehensive evaluation, ensuring that the model’s performance was not influenced by any specific data split.

## 3 Results

Table 1 presents the descriptive characteristics of the participants. Since the muscles near the Erector spinae are crucial for Adolescent Idiopathic Scoliosis (AIS), the electromyographic Root Mean Square (RMS) and PMSI indices of this region during natural standing posture are shown.

**TABLE 1:** Demographic data.

Variable	Value
Age	14.6 ± 2.7
Gender	46% female
Body Mass Index (BMI)	17.29 ± 1.23
Risser Sign	1.52 ± 1.21
Scoliosis curve	
Left curve	64.7%
Right curve	35.3%
Cobb Angle-T (°)	
Baseline	19.12 ± 5.95
Post treatment	
Cobb Angle-L (°)	
Baseline	15.28 ± 6.71
Post treatment	9.53 ± 4.21
ART-T (°)	
Baseline	8.97 ± 2.58
Post treatment	3.64 ± 2.10
ART-L (°)	
Baseline	4.36 ± 2.65
Post treatment	2.23 ± 1.97
SRS-22	
Baseline	3.25 ± 0.32
Post treatment	4.54 ± 0.26
BST (s)	
Baseline	88.34 ± 35.17
Post treatment	109.85 ± 43.67
Erector spinae RMS_concave_ (μV)	25.56 ± 7.83
Erector spinae RMS_convex_ (μV)	38.57 ± 7.43
Erector spinae PMSI	1.37 ± 0.19

Note: Cobb Angle -T: cobb angle of thoracic, Cobb Angle -L: cobb angle of lumbar, ART-T: thoracic angle of trunk rotation, ART-L: lumbar angle of trunk rotation, SRS-22: scoliosis - specific quality - of - life questionnaire, BST: Biering - Sorensen test.

### 3.1 Comparison of prediction models

This study compares the prediction performance of four models: the proposed TCN-LSTM hybrid model, the TCN model, the LSTM model, and a variant of Support Vector Regression (SVR) suitable for regression tasks.

**FIGURE 5:**
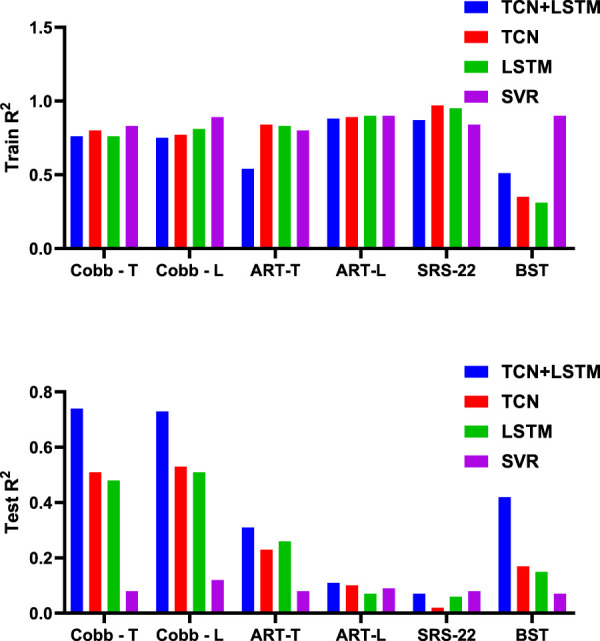
Mean value of R-squared for cross-validation of different models.

Table 2 and Figure 5 display the research results, revealing that prediction accuracy varies across different dependent variables. The SRS-22 test set had the lowest prediction accuracy, with all models failing to predict it effectively, with the highest accuracy (R^2^) not exceeding 0.21. The prediction accuracy for ART-L was also low, with a maximum of 0.22. In contrast, the prediction accuracy for the Cobb Angle was the highest, indicating that the Cobb Angle is more suitable for prediction using neural networks, whereas variables like SRS-22 and ART-L are less suitable.

**TABLE 2:** Mean value of R-squared for cross-validation of different models.

Time	TCN+LSTM		TCN		LSTM		SVR	
	Train R^2^	Test R^2^	Train R^2^	Test R^2^	Train R^2^	Test R^2^	Train R^2^	Test R^2^
Cobb Angle-T								
0 Week	0.65	0.63	0.76	0.55	0.81	0.60	0.87	0.07
24 Week	0.71	0.69	0.85	0.53	0.87	0.48	0.81	0.07
Total	0.76	0.74	0.80	0.51	0.76	0.48	0.83	0.08
Cobb Angle-L								
0 Week	0.63	0.61	0.66	0.58	0.65	0.53	0.87	0.12
24 Week	0.69	0.65	0.69	0.67	0.67	0.53	0.88	0.10
Total	0.75	0.73	0.77	0.53	0.81	0.51	0.89	0.12
ART-T								
0 Week	0.54	0.50	0.73	0.43	0.71	0.45	0.91	0.04
8 Week	0.55	0.32	0.67	0.25	0.63	0.28	0.82	0.09
16Week	0.76	0.12	0.84	0.11	0.83	0.08	0.84	0.10
24 Week	0.65	0.33	0.75	0.24	0.77	0.20	0.86	0.06
Total	0.54	0.31	0.84	0.23	0.83	0.26	0.80	0.08
ART-L								
0 Week	0.85	0.21	0.91	0.04	0.87	0.06	0.83	0.10
8 Week	0.86	0.14	0.82	0.13	0.80	0.09	0.85	0.11
16Week	0.81	0.15	0.88	0.12	0.84	0.11	0.87	0.07
24 Week	0.87	0.06	0.92	0.07	0.88	0.11	0.89	0.08
Total	0.88	0.11	0.89	0.10	0.90	0.07	0.90	0.09
SRS-22								
0 Week	0.94	0.02	0.97	0.01	0.98	0.05	0.88	0.11
8 Week	0.88	0.13	0.98	0.01	0.97	0.01	0.81	0.12
16Week	0.81	0.15	0.91	0.06	0.96	0.10	0.87	0.06
24 Week	0.89	0.21	0.98	0.01	0.95	0.05	0.83	0.07
Total	0.87	0.07	0.97	0.02	0.95	0.06	0.84	0.08
BST								
0 Week	0.54	0.43	0.32	0.23	0.35	0.26	0.86	0.10
8 Week	0.54	0.45	0.35	0.15	0.34	0.18	0.87	0.05
16Week	0.45	0.47	0.31	0.16	0.36	0.19	0.88	0.12
24 Week	0.52	0.43	0.45	0.12	0.47	0.16	0.89	0.06
Total	0.51	0.42	0.35	0.17	0.31	0.15	0.90	0.07

Note: Cobb Angle -T: cobb angle of thoracic, Cobb Angle -L: cobb angle of lumbar, ART-T: thoracic angle of trunk rotation, ART-L: lumbar angle of trunk rotation, SRS-22: scoliosis - specific quality - of - life questionnaire, BST: Biering - Sorensen test. The R-squares in the table are the mean of the cross-validated R-squares.

The comparison of models shows that SVR performed the worst in predicting all dependent variables, with the highest average accuracy on the test set not exceeding 0.12, and severe overfitting on the training set. The best-performing model was the TCN+LSTM hybrid model, followed by TCN and LSTM. The TCN+LSTM model outperformed the others by a large margin, consistently exceeding the accuracy of the other models across most tasks, while the difference between TCN and LSTM was minimal. Therefore, the following analysis focuses on the performance of the TCN+LSTM model in predicting Cobb Angle-T and Cobb Angle-L.

### 3.2 Prediction of Cobb angle

**FIGURE 6:**
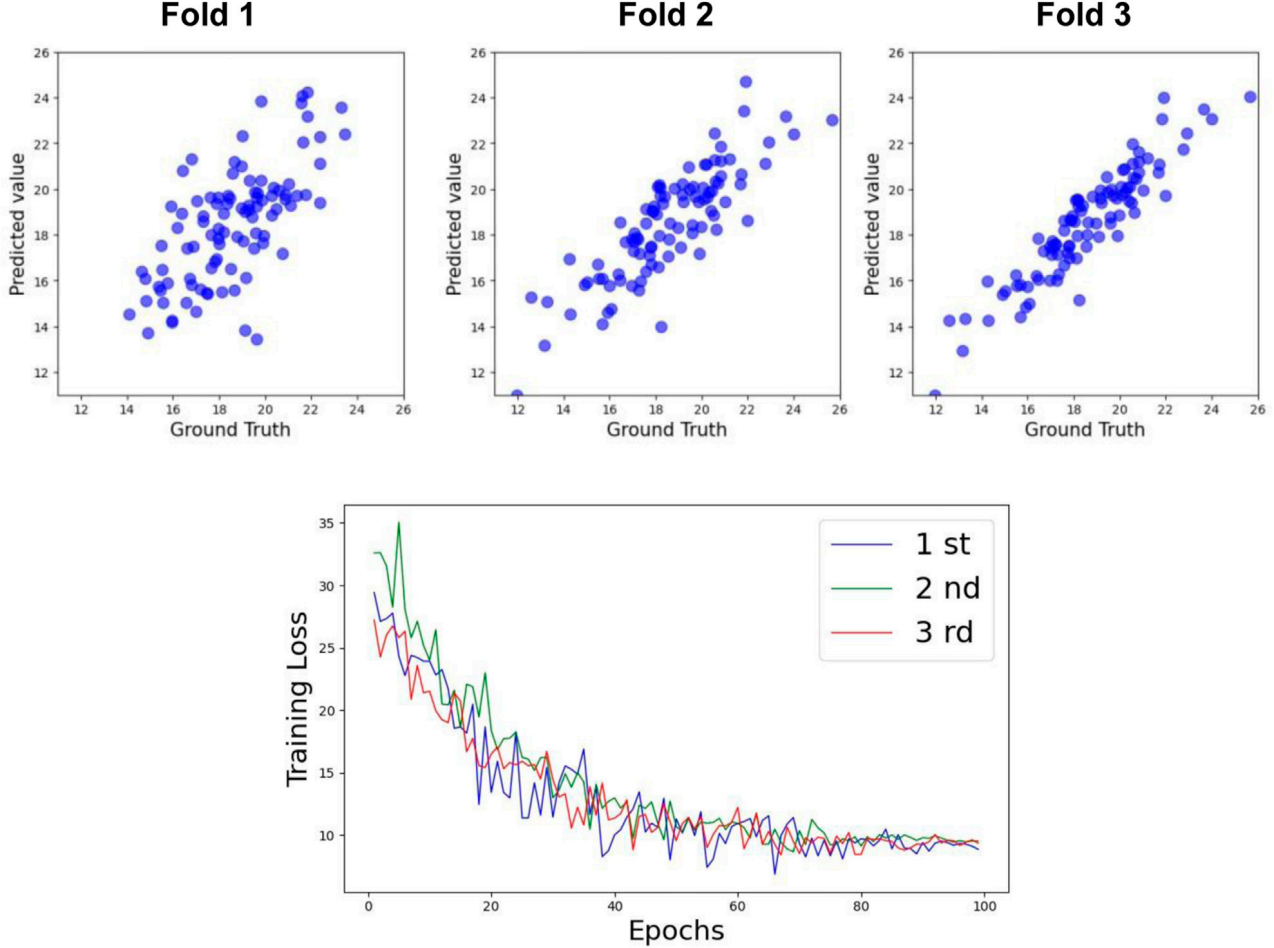
Performance of TCN+LSTM for predicting Cobb Angle-T.

Table 3 shows the prediction accuracy for Cobb Angle-T. The accuracy at Week 0 was relatively low (R^2^ = 0.63), whereas the accuracy at Week 24 was slightly higher (R^2^ = 0.69). The overall prediction accuracy across all samples was R^2^ = 0.74 for the test set. The scatter plots for the test set and the training loss function are shown in Figures 6, 8.

**TABLE 3:** Performance of TCN+LSTM in predicting Cobb Angle.

Time	TCN+LSTM			
	Train RMSE	Train R^2^	Test RMSE	Test R^2^
Cobb Angle-T				
0 Week	3.52 ± 0.43	0.65 ± 0.22	3.62 ± 0.22	0.63 ± 0.23
24 Week	3.20 ± 0.46	0.71 ± 0.15	3.33 ± 0.27	0.69 ± 0.11
Total	2.91 ± 0.34	0.76 ± 0.19	3.03 ± 0.32	0.74 ± 0.18
Cobb Angle-L				
0 Week	3.61 ± 0.26	0.63 ± 0.15	3.72 ± 0.31	0.61 ± 0.15
24 Week	3.31 ± 0.31	0.69 ± 0.18	3.52 ± 0.24	0.65 ± 0.21
Total	2.98 ± 0.29	0.75 ± 0.21	3.09 ± 0.22	0.73 ± 0.13

**FIGURE 7:**
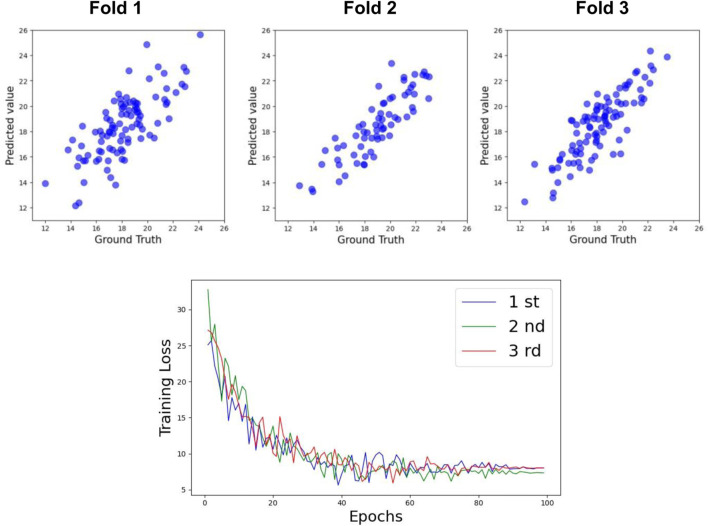
Performance of TCN+LSTM for predicting Cobb Angle-L.

**FIGURE 8:**
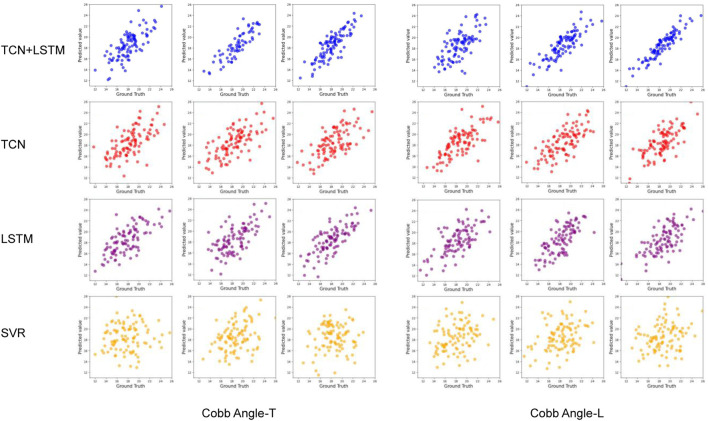
Scatter plots of cross - validation for predicting Cobb Angles using different models.

For Cobb Angle-L, as shown in Table 3, the accuracy at Week 0 was also low (R^2^ = 0.61), with a slightly improved prediction accuracy at Week 24 (R^2^ = 0.65). The overall test set prediction accuracy was R^2^ = 0.73. The scatter plots for the test set and the training loss function are presented in Figures 7, 8.

**FIGURE 9:**
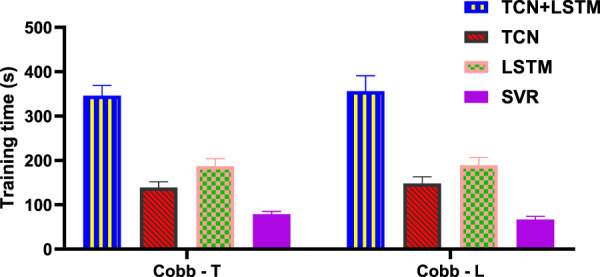
Training time for different models to predict Cobb angle.

The results in Figure 9 show that for the prediction of Cobb angle, the training time of TCN+LSTM is the longest, followed by that of LSTM and TCN, and finally SVR.

## 4 Discussion

This study demonstrates that the TCN-LSTM hybrid model achieves superior Cobb angle prediction accuracy (R^2^ = 0.63–0.74) compared to TCN, LSTM and SVR models when analyzing sEMG data from AIS patients, with SVR showing particular limitations due to overfitting (R^2^ = 0.07). The model’s effectiveness stems from TCN’s ability to capture temporal sEMG patterns combined with LSTM’s capacity for modeling long-term progression, offering significant potential to transform clinical practice by enabling more frequent, non-invasive monitoring of scoliosis progression. Beyond immediate clinical applications, this approach could facilitate the development of intelligent rehabilitation systems that provide real-time feedback during Schroth exercises, optimize treatment personalization, and reduce reliance on radiographic assessments. The technology’s framework may also be adaptable to other musculoskeletal disorders where muscle activation patterns correlate with disease progression. While the model shows particular strength in predicting structural spinal changes, its relatively lower accuracy for SRS-22 and ART-L measures highlights the complex multifactorial nature of these outcomes and suggests the need for complementary assessment methods in comprehensive patient care. These findings contribute to the growing field of AI-assisted rehabilitation by demonstrating how deep learning can extract clinically meaningful patterns from physiological signals (Du et al., 2024; Yao et al., 2023), with implications for both clinical decision-making and home-based monitoring solutions. Future research should focus on enhancing model generalizability and developing practical implementation strategies to maximize clinical impact.

This study demonstrates that the TCN-LSTM model effectively predicts Cobb angle progression in AIS patients undergoing Schroth therapy, with improving accuracy over time (R^2^ = 0.63–0.74) and decreasing RMSE values (3.52–2.91), indicating robust performance (Fei et al., 2023; Kriegeskorte and Golan, 2019). The model’s ability to correlate sEMG patterns with spinal curvature changes offers significant clinical potential beyond immediate predictions, enabling a paradigm shift in scoliosis management. By providing quantitative, real-time feedback on treatment response, this approach could revolutionize rehabilitation monitoring - reducing reliance on periodic radiographic assessments while enabling more dynamic treatment adjustments. The technology’s applications extend to tele-rehabilitation platforms, where continuous sEMG monitoring combined with predictive analytics could support remote patient care, particularly valuable for underserved regions. Furthermore, the framework established here may be adaptable to other musculoskeletal rehabilitation contexts where muscle activation patterns correlate with clinical outcomes. The consistent predictive performance across treatment phases suggests potential utility in both short-term intervention planning and long-term prognosis estimation, offering clinicians a powerful tool for personalized therapy optimization. These findings align with broader trends in precision rehabilitation, demonstrating how deep learning can extract clinically actionable insights from physiological time-series data (Fei et al., 2023; Kriegeskorte and Golan, 2019). Future implementations could integrate this technology with wearable sensors and mobile health platforms to create comprehensive monitoring ecosystems, though additional work is needed to standardize sEMG protocols across diverse clinical settings.

This study demonstrates that combining neural networks with sEMG data enables accurate prediction of Cobb angle changes during Schroth therapy in AIS patients, with prediction accuracy improving from R^2^ = 0.61 at baseline to R^2^ = 0.65 at week 24, and overall test set performance reaching R^2^ = 0.73. The progressive improvement suggests the model effectively learns temporal patterns from sEMG signals, offering clinically valuable insights for personalized treatment planning (Dolan et al., 2024). Beyond immediate clinical applications, this approach has transformative potential for scoliosis management by enabling continuous, radiation-free monitoring that could reduce reliance on frequent radiographs. The technology could be integrated into tele-rehabilitation platforms to support remote patient monitoring, particularly beneficial for geographically isolated populations. Furthermore, the framework established here may be adaptable to other musculoskeletal conditions where muscle activation patterns correlate with disease progression, potentially revolutionizing physiotherapy outcomes assessment across multiple domains. The demonstrated success in predicting structural spinal changes from sEMG data (Minotti et al., 2024) opens new possibilities for developing intelligent rehabilitation systems that combine wearable sensors with predictive analytics to optimize treatment protocols in real-time. While the current focus is on AIS, the methodology could be extended to adult degenerative scoliosis or other spinal deformities, providing a scalable solution for diverse patient populations. These advancements align with the growing trend toward precision rehabilitation medicine, where data-driven approaches enable more objective treatment evaluation and customization (Sitoula et al., 2015). Future implementations should focus on translating these technical achievements into clinically accessible tools while addressing practical challenges such as signal standardization across different patient demographics and clinical settings.

## 5 Conclusion

In this study, a hybrid model was constructed based on deep learning techniques, combining time-sequential convolutional networks (TCNs) and long-short-term memory networks (LSTMs), for predicting Cobb angle changes in adolescent idiopathic scoliosis (AIS) patients during Schrott training. By integrating basic patient characteristics and time-series electromyography (sEMG) data, the model was able to effectively capture the relationship between local short-term dependence and global long-term dependence. The experimental results showed that the TCN-LSTM model outperformed the traditional statistical model in predicting the spine angle changes in AIS patients with high accuracy and reliability, and the model prediction ability was further enhanced by calculating features such as the electromyographic symmetry index (PMSI) and the root mean square (RMS) value. The study provides accurate and personalised treatment support for AIS patients and provides data for the dynamic adjustment of clinical treatment plans. In addition, the study combines multi-dimensional metrics such as spinal rotation angle and muscular endurance, providing a more comprehensive assessment tool for clinical use. Overall, the study promotes the application of smart medical technology in AIS treatment, provides an innovative direction for early diagnosis and efficacy assessment, and has broad clinical significance and prospects.

## Data Availability

The original contributions presented in the study are included in the article/supplementary material, further inquiries can be directed to the corresponding author.
